# Does gradual weaning improve outcomes after successful Pavlik harness treatment in developmental hip dysplasia? A retrospective comparative study

**DOI:** 10.1007/s00402-026-06239-x

**Published:** 2026-02-18

**Authors:** Soner Kocak, Sabri Kerem Diril, Mustafa Caliskan, Gurkan Caliskan, Esref Terzi, Ali Ozyalcin, Sumeyra Dogan, Mehmet Akif Sahin, Adem Sahin, Abdulhamit Misir, Cemil Erturk

**Affiliations:** 1 Department of Orthopaedic and Traumatology, University of Health Sciences, Istanbul Kanuni Sultan Suleyman Training and Research Hospital, Istanbul, Turkey; 2 Department of Pediatric Radiology, University of Health Sciences, Istanbul Kanuni Sultan Suleyman Training and Research Hospital, Istanbul, Turkey; 3 Department of Orthopaedic and Traumatology, Diyarbakır Gazi Yaşargil Eğitim ve Araştırma Hastanesi, Diyarbakır, Turkey; 4 Department of Orthopedics and Traumatology, Bahcesehir University Medical School, Istanbul, Turkey

**Keywords:** Avascular necrosis, Developmental dysplasia of the hip, Frejka pillow, Pavlik harness, Recurrence, Treatment discontinuation

## Abstract

**Introduction:**

The Pavlik harness is the standard first-line treatment for developmental dysplasia of the hip (DDH) in infants younger than six months. However, the optimal strategy for discontinuation after successful hip reduction remains debated. This study aimed to compare clinical and radiological outcomes between immediate discontinuation and gradual weaning using the Frejka pillow following successful Pavlik harness treatment.

**Methods:**

We retrospectively analyzed data from 144 infants (166 hips) with DDH treated with the Pavlik harness between 2012 and 2023. Patients were categorized into two groups: Group A underwent immediate cessation, while Group B was gradually weaned using a Frejka pillow. Radiological follow-up included acetabular index (AI), lateral center-edge angle (LCEA), and assessment of AVN based on the Kalamchi-MacEwen classification, up to five years of age.

**Results:**

The two groups were demographically similar. There were no statistically significant differences in recurrence rates (Group A: 9%, Group B: 5%; *p* = 0.53) or AVN incidence (Group A: 6.25%, Group B: 10%; *p* = 0.55). Group B showed a non-significant trend toward higher LCEA values at five years (*p* = 0.08), potentially indicating better acetabular development. However, the slightly increased AVN rate in this group raises concerns regarding prolonged abduction.

**Conclusion:**

Both immediate discontinuation and gradual weaning protocols yield comparable outcomes in terms of recurrence and AVN. While gradual weaning may offer marginal benefit in acetabular development, the potential vascular risks warrant further investigation. Larger prospective studies are needed to establish standardized discontinuation guidelines in DDH management.

**Level of evidence:**

III, retrospective comparative study.

## Introduction

 Developmental dysplasia of the hip (DDH) is a common condition in infants, with a reported prevalence of approximately 1.4% according to recent meta-analyses [1]. Early diagnosis and treatment of the disease are crucial, as delayed intervention may lead to serious long-term complications such as chronic hip pain, osteoarthritis, limb length discrepancy, gait abnormalities, and joint contractures [2]. Ultrasound (US) is widely used for diagnosis, follow-up, shaping and terminating treatment [3].

The use of brace is considered the gold standard in babies under 6 months of age with DDH whose hips are reduced [4–6]. Treatment of DDH with Pavlik harness has successful and good results in the short and long term [7, 8]. There are also dynamic splints other than pavlik harness, such as Frejka Pillow and and the purpose of dynamic splints is to encourage dynamic reduction of the hip in the treatment of DDH [6]. Complications such as avascular necrosis (AVN) and femoral nerve paralysis may develop with splint-based treatments, even when properly applied. Importantly, extended use of the Pavlik harness has been specifically linked to an increased risk of AVN [6, 9, 10].

There is no consensus among orthopedic surgeons regarding discontinuation of treatment in DDH cases treated with Pavlik harness [11]. In order to minimize possible complications, Pavlik harness and other dynamic braces may need to be used for as little time as possible. However, studies in this field remain limited, and long-term outcomes are still uncertain. The aim of this study is to compare the clinical and radiological outcomes of immediate discontinuation of Pavlik harness treatment versus gradual weaning using the Frejka pillow in infants who achieved successful reduction with Pavlik harness.

## Methods

### Study design and patient selection

**Fig. 1:**
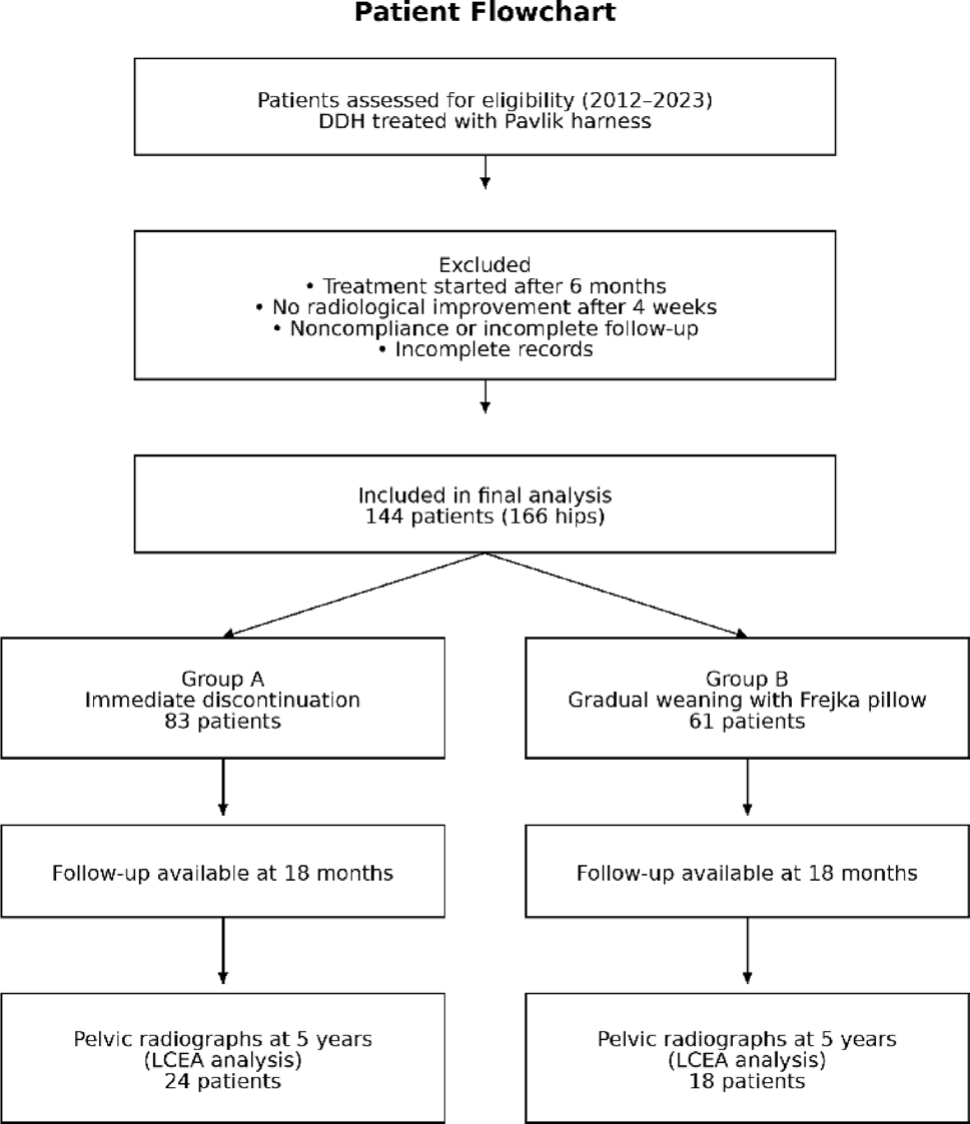
Patient flowchart of the study. Flow diagram illustrating patient selection, exclusion criteria, group allocation, and follow-up throughout the study period

Patients diagnosed with developmental dysplasia of the hip and treated with the Pavlik harness between January 2012 and January 2023 at our clinic were retrospectively evaluated. Data were collected from hospital records. When medical records were incomplete, structured telephone interviews with caregivers were conducted during routine outpatient visits to verify treatment adherence, follow-up attendance, and developmental milestones. Only patients with complete medical documentation who met the predetermined inclusion criteria were enrolled in the study. Patient selection, exclusion criteria, and group allocation are summarized in the patient flowchart (Fig. 1).

### Inclusion and exclusion criteria

Patients were included if they were younger than 6 months at the initiation of treatment, had hip dysplasia classified as Graf type IIb or higher on ultrasonographic evaluation, had received no prior treatment, and demonstrated both clinical and radiological normalization following consistent and appropriate use of the Pavlik harness. Additional inclusion criteria included adherence to the treatment protocol, a minimum follow-up duration of 18 months, normal muscle function, and achievement of age-appropriate developmental milestones [12, 13]. 

Exclusion criteria included teratologic hip dislocation, neuromuscular disorders, overweight infants unable to tolerate the harness, noncompliance with treatment or follow-up, lack of radiological improvement after 4 weeks of correct Pavlik harness use, initiation of treatment after 6 months of age, and incomplete medical records that could not be verified. Patients who showed no improvement after 4 weeks of appropriate Pavlik harness application (≥ 23 h/day) were discontinued from harness treatment to prevent Pavlik disease and were excluded from the study.

### Treatment protocol

All patients underwent the same treatment protocol using a Pavlik harness. Ultrasonographic evaluations were performed at regular intervals during treatment. In patients demonstrating significant but incomplete improvement after 4 weeks, the Pavlik harness was continued for an additional 4 weeks with repeat ultrasonographic assessment. Treatment was considered successful when follow-up ultrasonographic evaluations demonstrated an alpha angle greater than 60 degrees, accompanied by normalization or improvement in beta angle, and a stable clinical examination including negative Barlow and Ortolani tests with no abduction limitation. Treatment discontinuation was based on radiological normalization, defined as an alpha angle greater than 60°, rather than on a predefined number of ultrasonographic examinations. Accordingly, the exact number of ultrasonographic assessments performed before Pavlik harness discontinuation was not standardized and may have varied between patients. Radiological normalization was confirmed primarily by ultrasonography up to 6 months of age, while anteroposterior pelvic radiographs were obtained after 6 months in borderline or late-presenting cases [11–13]. 

### Group allocation and weaning strategy

Following successful Pavlik harness treatment, patients were allocated into groups according to the routine clinical practice of two different pediatric orthopedic surgeons. Group allocation was determined at the time of treatment and was not influenced by patient characteristics or disease severity.

**Fig. 2:**
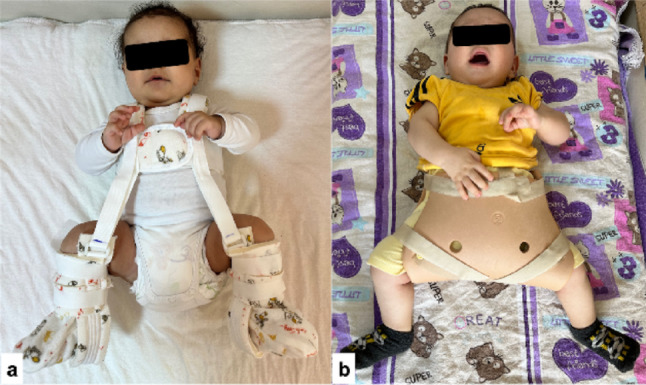
Clinical application of abduction orthoses in the treatment of developmental dysplasia of the hip. a Pavlik harness application demonstrating dynamic positioning of the hips in flexion and abduction, allowing functional reduction while preserving active motion. b Frejka pillow application showing abduction positioning, used during the gradual weaning phase following initial successful treatment

In Group A, the Pavlik harness was discontinued immediately after radiological normalization was achieved. In contrast, in Group B, despite confirmed radiological normalization, treatment was gradually weaned using a Frejka pillow for a total of 3 months: continuous use during the first month, followed by nighttime use (approximately 12 h per day) for the subsequent two months (Fig. 2). This weaning protocol was based on our institutional practice guidelines and clinical experience, aiming to maintain hip stability while gradually reducing external support. Although not universally standardized, similar weaning durations have been reported in previous observational studies involving dynamic abduction orthoses [6, 14, 15]. 

### Radiological evaluation and follow-up

Radiographic follow-up was performed at 6, 9, 12, 15, and 18 months of age. Evaluations included assessment of the Shenton–Menard line, acetabular index (AI), and femoral head ossification center position according to the Tönnis classification [16]. Recurrence was documented and managed according to standard treatment escalation protocols, with patients analyzed according to their original group allocation. Avascular necrosis (AVN) was assessed using the Kalamchi–MacEwen classification [17]. When available, the lateral center-edge angle (LCEA) [18] was measured on anteroposterior pelvic radiographs obtained at 5 years of age [19]. 

### Blinding and reliability

To minimize observer bias, all ultrasonographic and radiographic examinations were performed and reviewed by a pediatric radiologist blinded to treatment allocation. Radiological measurements were independently evaluated by two observers, and interobserver reliability was assessed using intraclass correlation coefficients. Demographic characteristics—including sex, age, side affected, ethnicity, and associated risk factors—were documented and analyzed to ensure group comparability. Post hoc corrections were then performed using multivariate logistic regression to account for possible confounders such as treatment initiation age, sex, laterality, and baseline hip severity categorized by Graf classification. Although the study lacked randomization, methodological rigor was maintained through comprehensive data analysis to reduce potential bias.

### Statistical analysis

Data analysis was carried out with R software. The normality of the data distributions was assessed prior to analysis, and appropriate statistical tests were selected accordingly. Paired t-test and Unpaired/Welch t-test were applied to determine the significance level of the relationship between numerical variables. Associations between categorical variables were assessed using the Chi-square test, with statistical significance defined as *p* < 0.05.

## Results

### Patient characteristics

A total of 144 patients (166 hips) were included in the study, with 83 patients in Group A and 61 patients in Group B. The patient selection process and final cohort distribution are illustrated in Fig. 1. The distribution of sex, laterality, ethnicity, and baseline clinical characteristics was comparable between groups, with no statistically significant differences observed in demographic variables (Table 1).

**Table 1:** Comparative data and demographic characteristics of patients

	Group A (%)	Group B (%)
Gender		
Male	21 (25)	17 (28)
Female	62 (75)	44 (72)
Ethnicity		
Asian	69 (83)	48 (78)
White European	11 (13)	12 (20)
Other	3 (4)	1 (2)
Laterality		
Right	24 (29)	16 (26)
Left	46 (55)	36 (59)
Bilateral	13 (16)	9 (15)
Risk factors		
Family history	25	14
Breech presentation	20	17
Multiple birth	1	3
Pre-treatment alpha angle (degree);		
50–59	36 (38)	27 (39)
43–49	53 (55)	33 (47)
< 43	7 (7)	10 (14)
Age at treatment initiation (weeks)	12.1	11.3
Pavlik harness treatment duration (weeks)	8.7	9.3
Follow-up duration (month)	35.2 [range 18–69]	40.5 [range 18–81]

### Treatment groups and follow-up

The mean age at treatment initiation (weeks) was 12.1 (range 4–22, median 12) in Group A and 11.3 (range 4–21, median 12) in Group B. There was no difference between Group A and Group B in age at treatment initiation (weeks) and these values were statistically similar (*p* = 0.32). The duration of Pavlik harness use was similar between the two groups, indicating comparable baseline treatment exposure (*p* = 0.39) (Table 1). All patients included in the analysis achieved both radiological and clinical normalization following Pavlik harness treatment prior to group allocation.

### Radiological outcomes

Pre-treatment alpha angle and acetabular index (AI), did not differ significantly between groups. At 18 months of follow-up, AI values showed improvement in both groups, with no statistically significant intergroup difference.

Pelvic radiographs obtained at 5 years of age were available for a subset of patients in both groups, including 24 patients (28.9%) in Group A and 18 patients (29.5%) in Group B. In this subgroup analysis, Group B showed higher lateral center-edge angle (LCEA) values than Group A; however, this difference did not reach statistical significance at the conventional level (*p* = 0.08) (Table 2).

**Table 2:** Mean radiological values

	Group A	Group B	*p*-value
Pre-treatment alpha angle	48.7º (38–59)	47.8º (36–58)	0.26
AI before treatment	37.6º (26–47)	38.1º (27–49)	0.53
AI (18 Months)	24.8º (15–39)	23.7º (14–36)	0.17
LCEA (5 years old)	23.2º (17–31)	25.6º (18–34)	0.08

AI, acetabular index; LCEA, lateral center edge angle

### Complications and recurrence

During follow-up, AVN was identified in 6 hips in Group A (3 Grade I, 2 Grade II, and 1 Grade III), representing an incidence of 6.25%, whereas Group B showed AVN in 7 hips (2 Grade I, 2 Grade II, 2 Grade III, and 1 Grade IV), corresponding to an incidence of 10%. Although both the frequency and severity of AVN were numerically higher in Group B, the difference in overall incidence between the groups was not statistically significant (*p* = 0.55) (Table 3).

**Table 3:** Patients with avascular necrosis during follow-up

	Group A (%)	Group B (%)
Total hip	96	70
Follow-up duration (month)	35.2 [range 18–69]	40.5 [range 18–81]
Avasculer necrosis		
Grade I	3 (3)	2 (3)
Grade II	2 (2)	2 (3)
Grade III	1 (1)	2 (3)
Grade IV	0	1 (1)

Recurrence of hip dysplasia was observed in both groups during follow-up, with a slightly higher proportion in Group A than in Group B; however, the difference was not statistically significant. All recurrent cases were managed according to standard treatment escalation protocols (Table 4). No cases of femoral nerve palsy were recorded, and no other serious or untreatable complications occurred.

**Table 4:** Recurrence and treatment summary after Pavlik Harness application

	Total patients	Recurrence (n, %)	Treatment methods	Statistical significance (p-value)
Group A	91	8 (9%)	2 - Static abduction brace 3 - Closed reduction + casting under anesthesia 3 - Open reduction + casting under anesthesia	*p* > 0.05 *p* = 0.53
Group B	64	3 (5%)	1 - Static abduction brace 1 - Closed reduction + casting under anesthesia 1 - Open reduction + casting under anesthesia	

## Discussion

The Pavlik harness is a well-established and effective treatment for infants under six months of age with DDH. Our findings indicate that whether treatment was discontinued abruptly or gradually with the use of a Frejka pillow after normalization, subsequent hip development and the incidence of complications such as recurrence or AVN were not significantly affected.

A recent meta-analysis identified female sex, breech presentation, and a positive family history as major risk factors for DDH, whereas prematurity appeared to confer a protective effect [20]. In our cohort, the comparable distribution of these variables between groups strengthens the internal validity of our results and minimizes potential confounding. Numerous studies emphasize that the age at treatment initiation plays a decisive role in the success of Pavlik harness therapy [12, 21]. By restricting inclusion to infants diagnosed before six months, we controlled for this factor, ensuring comparability across groups, where treatment initiation age and pre-treatment alpha angles were statistically similar.

Complications following Pavlik harness therapy include treatment failure, recurrence, AVN, femoral nerve palsy, and late acetabular dysplasia [22, 23]. Outcomes are strongly influenced by age at initiation, hip type per Graf classification, and treatment duration [24, 25]. To ensure validity, only patients who achieved normal hip values with Pavlik harness treatment were included, while non-responders were excluded. This homogeneity allowed for more accurate comparison of epidemiological and clinical risk factors, and indeed, our analysis revealed no significant differences between groups in treatment age, alpha angles, or duration, enhancing reliability. Because only hips that achieved radiological normalization following Pavlik harness treatment were included, baseline Graf classification was relatively homogeneous across the study cohort. Stratified outcome analyses by initial Graf type were therefore not performed, as further subdivision would have resulted in small subgroup sizes with limited statistical interpretability.

Consensus guidelines recommend discontinuation of Pavlik harness once radiological normalization is achieved, usually after 6–8 weeks of treatment, though no maximum duration is defined as long as the device is tolerated [25]. In our cohort, mean treatment duration exceeded eight weeks in both groups, with cessation only after normalization. Despite these guidelines, there remains no uniform agreement on the optimal duration or precise discontinuation criteria among pediatric orthopedic specialists [11, 26]. Some clinicians extend treatment by introducing a Frejka pillow, a practice that persists despite limited supporting evidence. Our study specifically compared immediate cessation with gradual weaning, finding no statistically significant differences in long-term outcomes.

The Frejka pillow represents an active abduction orthosis and inherently prolongs hip abduction following radiological normalization. This difference in abduction exposure was intentional and reflects the central clinical question of the present study: whether extending abduction after successful Pavlik harness treatment provides additional benefit. Despite the longer duration of abduction in the gradual weaning group, no statistically significant improvement in recurrence or complication rates was observed, suggesting that routine prolongation of abduction after normalization may not be necessary in all cases.

Gradual weaning after ultrasonographic normalization is often adopted based on theoretical concerns regarding residual instability during acetabular remodeling rather than on strong evidence. Our results demonstrate that when normalization is confirmed on consecutive ultrasonographic examinations, immediate discontinuation does not increase recurrence or complication rates compared with gradual weaning. From a health economics perspective, gradual weaning prolongs treatment duration and increases indirect costs and caregiver burden.

Recurrence of dysplasia remains a clinical concern, especially in patients with positive family history, breech presentation, or delayed initiation of treatment [27]. In our study, recurrence rates were slightly higher in the immediate cessation group (9%) compared with the gradual group (5%), though the difference was not statistically significant. These findings support the view that recurrence is multifactorial and not necessarily influenced by the method of discontinuation.

Patients were assessed for AVN using direct radiography at 18 months. Although a higher rate of radiologically detected AVN was observed in the gradual cessation group, the difference did not reach statistical significance. Daniel et al. [15] reported similar findings in children over 12 months, where gradual termination was associated with increased AVN rates compared to abrupt cessation. While these results suggest a potential trend, our retrospective and non-randomized design introduces limitations. Group allocation was based on surgeon preference, which creates selection bias and confounding by indication. Although post hoc multivariate adjustment was applied, residual confounding cannot be completely excluded. It is generally recognized that AVN and femoral nerve palsy are associated with the use of Pavlik harness beyond the safe zone [10, 28], yet prolonged use of abduction orthoses may also elevate AVN risk. Li et al. further confirmed the association between delayed treatment, AVN, and unsatisfactory hip function in late-diagnosed cases treated by open reduction, with older age at surgery, AVN, and more extensive operations as independent predictors of poor outcomes [29]. Our results reinforce the critical importance of early intervention and suggest that early reduction combined with timely discontinuation may help preserve long-term hip function. The importance of early diagnosis is underscored by Wicart et al., who reported significant shortcomings in early clinical screening and recommended systematic ultrasound protocols [30]. In contrast, our cohort benefited from routine ultrasound before six months, enabling early diagnosis, timely Pavlik harness initiation, and structured discontinuation. This proactive approach likely explains the comparable recurrence and AVN rates across both cessation strategies, further highlighting the value of systematic early detection.

Despite early treatment, residual acetabular dysplasia may persist, predominantly affecting the acetabulum and predisposing patients to premature osteoarthritis later in life [31]. In our study, no significant differences in AI values were observed during the early post-treatment period. However, hips were reassessed at age five using LCEA measurements, in line with recommendations that follow-up extend at least to age five to enable detection of late acetabular dysplasia [32, 33]. As the child matures, ultrasound assessment gives way to radiographic evaluation, shifting from AI to LCEA as the primary parameter [34]. In our subgroup analysis, marginally higher LCEA values were observed in the gradual weaning group (*p* = 0.08), raising the possibility of a protective effect against late dysplasia. However, this analysis was based on a small sample size (24 hips in Group A and 18 in Group B), increasing the risk of attrition bias and limiting generalizability. Given the exploratory p-value threshold (*p* < 0.10), these findings should be interpreted cautiously but may signal clinically relevant trends worthy of further prospective validation. Rampal et al. similarly demonstrated that residual dysplasia often persists despite successful reduction and reported that modified Dega acetabuloplasty can correct this deformity while minimizing the risk of acetabular retroversion [35]. In contrast, our non-surgical cohort treated with Pavlik harness followed by either immediate or gradual cessation demonstrated comparable early acetabular outcomes, though the slightly higher LCEA values in the gradual group suggest potential long-term benefits that merit further study.

The importance of fostering global collaboration and shared expertise in the management of DDH has been highlighted by a recent editorial, emphasizing that enhanced understanding of disease variability and treatment paradigms can lead to improved patient outcomes [36]. In this context, our study contributes robust, comparative data on cessation strategies (immediate discontinuation versus gradual Frejka weaning) that may inform evidence-based guidelines across diverse clinical settings.

This study has several limitations. The retrospective, non-randomized design introduces potential selection bias, and although multivariate adjustment was performed, residual confounding cannot be excluded. Attrition bias—particularly in the 5-year LCEA analysis—limits the strength of long-term outcome assessment. In addition, the absence of statistically significant differences between groups may partly reflect limited statistical power. Given the modest effect sizes observed, the study may have been underpowered to detect small but clinically relevant differences, and the possibility of a type II error cannot be excluded. Although baseline hip severity according to the Graf classification was included in multivariate analyses, the sample size did not permit detailed stratified comparisons by initial Graf type and treatment group; therefore, the findings should be interpreted as hypothesis-generating. Furthermore, the single-center design and relatively homogeneous cohort limit generalizability. Treatment-related confounding may also exist, as the Frejka pillow provides continued hip abduction, resulting in inherent differences in abduction exposure between groups. Finally, treatment discontinuation was based on radiological normalization rather than a standardized number of ultrasonographic examinations, which may have introduced surveillance bias. A formal cost-effectiveness analysis was not performed.

## Conclusion

In this study, no statistically significant difference was found between immediate cessation and gradual weaning of Pavlik harness treatment in terms of recurrence and AVN rates. However, the proportionally higher AVN rate in the gradually weaned group suggests that prolonged use of abduction devices may increase the risk of complications. Although higher LCEA values observed in this group may indicate a protective effect against late acetabular dysplasia, limited long-term follow-up restricts definitive conclusions. These findings highlight the need for prospective multicenter studies with expanded cohorts and greater demographic heterogeneity to guide evidence-based decisions on treatment discontinuation in DDH.

## Data Availability

All data from this study are available upon reasonable request to the corresponding author.

## **Declarations**

### Artificial intelligence

ChatGPT (OpenAI, USA) was used exclusively to assist with language and grammatical refinement. All suggested edits were carefully reviewed and approved by the authors, who take full responsibility for the scientific content.
